# Exploring purine analogues as inhibitors against Katanin, a microtubule severing enzyme using molecular modeling approach

**DOI:** 10.1038/s41598-024-83723-7

**Published:** 2024-12-30

**Authors:** Vibhuti Saxena, Pruthanka Patil, Purva Khodke, Bajarang Vasant Kumbhar

**Affiliations:** Department of Biological Sciences, Sunandan Divatia School of Science, SVKM’s Narsee Monjee Institute of Management Studies (NMIMS) Deemed-to-be University, Vile Parle (West), Mumbai, 400056 Maharashtra India

**Keywords:** Katanin, Microtubule, Purine analogues, Structure-based drug design, Molecular dynamic simulations, Biochemistry, Biophysics, Chemical biology, Computational biology and bioinformatics, Drug discovery, Molecular medicine, Cancer, Cancer prevention, Cancer therapy

## Abstract

Katanin, a key protein in cellular architecture, plays a crucial role in severing microtubules, which are vital components of the cytoskeleton. Given its central involvement in cell division and proliferation, katanin represents a promising target for therapeutic intervention, particularly in cancer treatment. Inhibiting katanin’s function could potentially hinder the uncontrolled growth of cancerous cells, making it an attractive target for novel anti-cancer therapies. Previous studies have shown that purine-based compounds exhibit a strong affinity for microtubule-severing enzymes. In this study, we aim to identify potential purine-type inhibitors of katanin using molecular modeling techniques. A total of 276,280 purine-type compounds from the PubChem database were subjected to structure-based high-throughput virtual screening, followed by ADME prediction, PASS analysis, and molecular docking studies. These efforts led to the identification of two potent compounds: PubChem CID 122589735 and 123629569, which demonstrated strong binding interactions with katanin. Molecular dynamics simulations further revealed that these compounds effectively altered katanin’s conformation when compared to ATP. Additionally, binding energy calculations indicated that PubChem CID 122589735 exhibited the strongest binding affinity for katanin, with the binding free energy ranking as follows: 122589735 > 123629569 > ATP. Our findings suggest that the screened compounds, particularly PubChem CID 122589735, hold promise as potential katanin inhibitor. These compounds could play a significant role in the development of new anti-cancer therapies targeting a variety of carcinoma. Future research, including in vitro and in vivo studies, is essential to assess the efficacy and safety of these inhibitors, paving the way for innovative cancer treatments.

**Supplementary Information:**

The online version contains supplementary material available at 10.1038/s41598-024-83723-7.

## Introduction

**Fig. 1:**
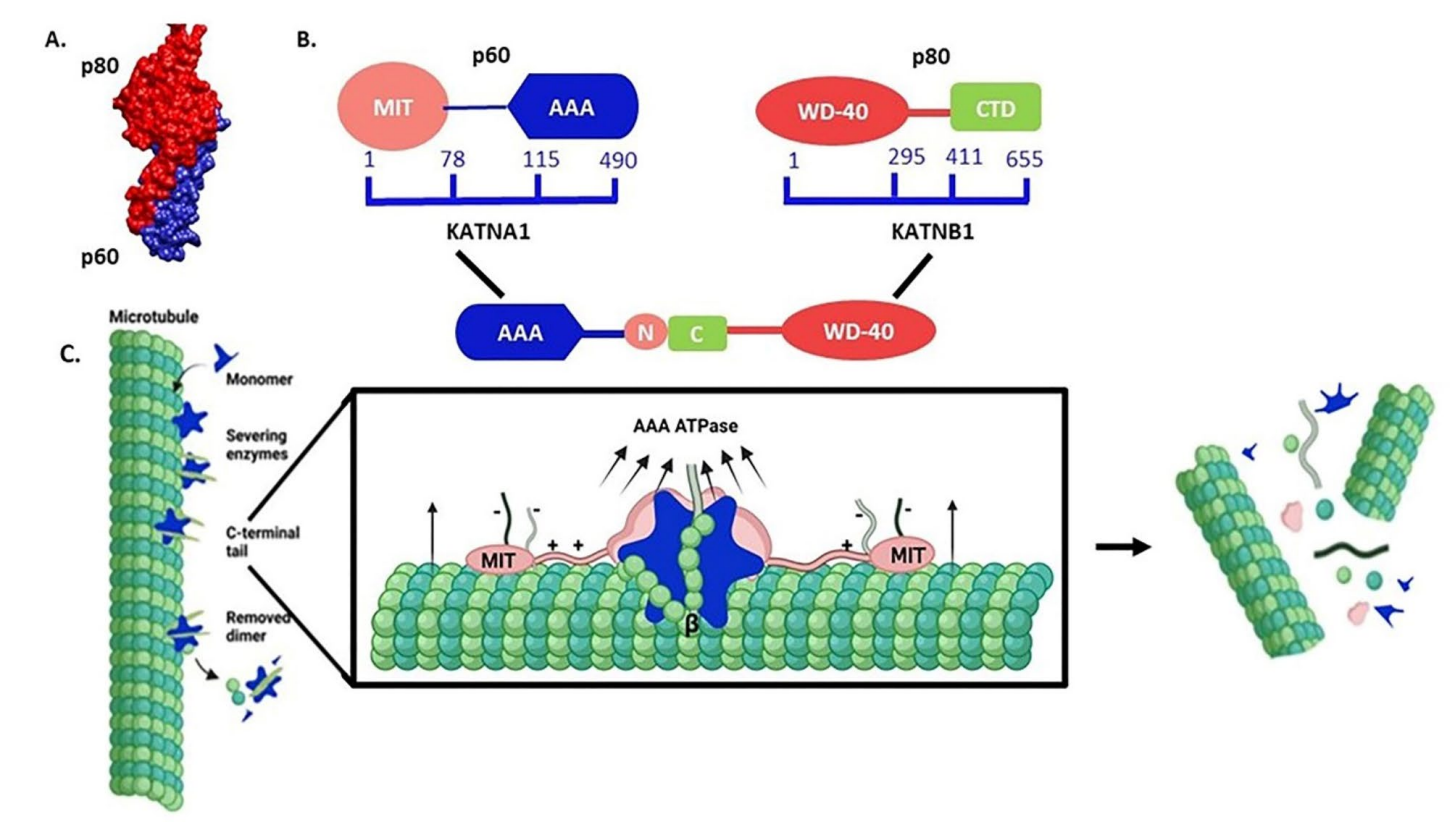
Schematic representation of katanin and its mechanism of microtubule severing. Here, **(A)** depicts the katanin complex, consisting of two subunits: the p80 subunit (red) and the p60 subunit (blue), p60 is responsible for microtubule severing while p80 is responsible for loading of p60 subunit. **(B)** Shows the different domain of katanin p60 such as N-terminal MT-interacting and trafficking (MIT) domain which forms a crucial interaction with the C-terminal domain (p80-CTD) of the p80 subunit. Next, the C-terminal AAA domain of the p60 subunit is responsible for MT-severing functions which requires the formation of a hexameric structure and the hydrolysis of ATP to ADP and inorganic phosphate (Pi) for the force generation. **(C)** Shows the schematic representation of the monomeric katanin complex assembling to form a hexamer around the C-terminal tail of the tubulin (green). This interaction triggers the pulling and severing of the microtubule, as the AAA domain uses ATP hydrolysis to deform tubulin and weaken interdimer connections, ultimately releasing tubulin from the microtubule.

Microtubules (MTs) are filamentous proteins that serve as essential components of the cytoskeleton, playing critical roles in mitosis, intracellular transport, cell signalling, and cell shape^1^. The polymerization of α tubulin and β tubulin heterodimeric subunits forms MTs. The regulation of MT dynamics involves numerous microtubule-associated proteins which include tau^2^, MAPs, MARKs^3^, and microtubule-severing enzymes such as katanin, spastin, and fidgetin^4^. Among these regulators, the microtubule-severing enzyme, katanin plays a crucial role in the fragmentation and reorganization of MTs (Fig. 1), facilitating the generation of new microtubule ends and promoting their remodelling within the cellular environment^5^. Katanin was identified as the first microtubule-severing enzyme^6^. It is AAA-ATPase heterodimeric protein and is made up of the catalytic p60 subunit (KATNA1) and regulatory p80 subunit (KATNB1)^7,8^ as shown in Fig. 1. The acronym “AAA” stands for the family’s distinctive conserved amino acid sequence of the ATPase domain, which repeats the amino acids alanine, and arginine^9^. The catalytic p60 subunit has microtubule-stimulated ATPase and severing activity^10^. The p60 subunit is attracted to the site of the microtubule severing by the p80 subunit, which functions as the regulatory component and interacts with microtubules^11^. MTs can be severed by the ATPase subunit p60 alone by ATP hydrolysis^6^, but the p80 subunit makes this activity more effective^8^. Katanin remains in a monomeric state at cellular concentrations and katanin hexamerization is essential for severing activity. Katanin hexamerization and the presence of tubulin C-terminal tails are necessary for severing to occur^11^.

Katanin severs microtubules through the progressive removal of tubulin subunits^12^, and katanin achieves this by repetitively pulling on the C-terminal tails of tubulin subunits from the MTs^11^. The ATP hydrolysis-driven conformational changes of the hexameric ring enable it to exert force or produce motion that helps to remove the tubulin subunit from the MT^13^. Recently, discrete lattice-based Monte Carlo models incorporating microtubule (MT) dynamics and severing enzyme activity have been developed to elucidate the effects of severing enzymes on tubulin mass, MT quantity, and MT length^14^. However, the breaking and reorganization of MTs by the katanin play a crucial role in ciliogenesis^15^, cell size regulation^4^, plant cell wall biosynthesis and phototropism^16,17^. Besides, the mutation in the katanin subunit leads to neurodegenerative disorders^18,19^. The absence of katanin expression is implicated in various conditions, including ciliopathies^15,20^, impaired corticogenesis and spermiogenesis^21–23^.

Moreover, increased expression of katanin has been observed in non-small cell lung cancer (NSCLC), specifically correlating with lymph node metastasis^24^. Also, elevated levels of katanin contribute to enhanced cell proliferation and migration in primary breast cancer tissue^25^. Likewise, in prostate cancer, the expression of katanin p60 enhances the migratory capacity of cells^26,27^. While, in the case of papillary thyroid carcinoma, there is a correlation between katanin expression and worsened tumor characteristics^28^. Hence, katanin is an important target for designing a potential anticancer agent against numerous carcinomas.

Consequently, previous studies have shown that purine-type compounds induce microtubule fragmentation and promote lung cancer cell death through interactions with katanin^29^. Similarly, Gao and colleagues have designed, synthesized, and evaluated the biological activity of novel diaryl substituted fused heterocycles as dual ligands targeting tubulin and katanin^30^. Moreover, triazolopyridine-based fragments have activity against the mutant forms of katanin^31^. Thus, the ability of purine inhibitors to effectively target katanin makes them promising candidates for drug development. However, effective medications targeting katanin for treating various types of carcinomas are still unavailable.

Hence, the present study aimed to identify the potential katanin inhibitors utilizing the purine-type compound library from the PubChem database, further employing the virtual screening, PASS biological activity prediction, ADME-T properties prediction, molecular docking, and molecular dynamics simulation. The application of these computational techniques facilitated the expeditious identification and characterization of potential katanin inhibitors within a vast chemical repository. By streamlining the process, these computational methodologies significantly reduced the time and resource expenditure typically associated with traditional experimental approaches for identification of potential inhibitors, as employed in the earlier study^32,33^. Ultimately, the MD simulations served as a robust validation tool for computational drug discovery^34–36^. Through these concerted efforts, our investigation successfully identified promising purine-type inhibitors, thereby paving the way for the development of novel therapeutic strategies targeting katanin for numerous carcinomas.

## Computational methodology

### Preparation of katanin structure and purine-type drug library

The crystal structure of human katanin (PDB ID: 5ZQM.pdb)^37^ was retrieved from RCSB Protein Data Bank. The missing residues in the crystal structure of katanin from 281 to 284, 314–319, 341–348, and 400–402 were modeled using the ‘structure editing’ tool of Chimera v1.18^38^ through Modeller. The refined 3D structure of katanin was further utilized for the virtual screening, molecular docking and molecular dynamics simulation. Next, to identify a potential katanin inhibitor, purine type compounds were retrieved from the NCBI PubChem compound database^39,40^, yielding a total of 2,76,280 purine-type compounds, the workflow of the selection of purine type compounds are mentioned in the Supplementary Fig. 1. These purine-type compounds existed in Structured Data File (SDF) format, which were converted to PDBQT file format using OpenBabel 3.1.1 software^41^ for virtual screening and hit identification.

## Structure-based virtual screening and hit identification

High-throughput Virtual screening was performed using AutoDock Vina 1.2.0, a methodology described by Trott and Olson^42^. It uses a newer and more accurate scoring function called “Vina”, and screens large libraries of compounds to identify potential hits for further development. Recognizing the crucial role of the ATP binding pocket in katanin’s function, we strategically designated this region as the target site for virtual screening. Subsequently, we used InstaDock v1.0^43^ software, a tool designed to streamline the analysis of docking results generated by QuickVina-W or AutoDock Vina 1.2.0. It filters the ‘docked out files,’ according to their binding affinity values, and extracts specified number of top hits. Notably, the preference for AutoDock Vina 1.2.0 over the conventional AutoDock stems from its demonstrably superior accuracy and processing speed^42^. Finally, we meticulously selected the identified hit compounds based on their exceptional binding energies, signifying their predicted strong affinity for the katanin target. The most promising five compounds were then ushered forward for a comprehensive evaluation of their biological properties, physicochemical characteristics, and pharmacokinetic profiles.

## Pass prediction and ADMET

The top five purine-type compounds underwent assessment for their biological characteristics through the Way2drug web server^44^. The accuracy of the biological activity predictions depends on the Pa (probability of activity) and Pi (probability of inactivity) values^44^. If a compound exhibits a Pa value that surpasses its corresponding Pi value, it is deemed to be biologically active^45^. This elevated Pa score indicates the potential for the compound to elicit the anticipated pharmacodynamic effects, as inferred from the algorithmic predictions. This diverse method offers a thorough evaluation of potential drug characteristics, encompassing pharmacokinetics and toxicity, aiding informed choices during drug exploration and advancement^44^. Next, For ADME-T properties prediction, Swiss-ADME server^46^ and pkCSM web server^47^ were used to assess the physicochemical and pharmacokinetic properties of a chosen set of compounds. It effectively identified properties like drug similarity, solubility, lipophilicity, bioavailability and toxicity.

## Molecular docking of katanin and purine-type compounds

To obtain energetically favourable conformation of selected purine-type compounds with katanin, molecular docking was conducted through AutoDock4.2.3^48^. The AutoDock employs a scoring function based on the AMBER force field, which provides better estimates for binding affinities^49^. The grid was positioned over the ATP binding pocket utilised in above-mentioned virtual screening process, to define the docking region. Control docking of ATP with katanin was also performed. The AGS, an ATP analogue bound with katanin (PDB ID: 5ZQM.pdb), was changed to ATP using Discovery Studio Visualizer^50^ for molecular docking. Compounds with the lowest binding energies were then used for molecular dynamics simulations. Further, the bonded and non-bonded intermolecular interactions of most energetically favourable conformations with katanin were analysed using the BIOVIA Discovery Studio software^50^.

## Molecular dynamics simulations

To explore the refined binding mode and affinity of katanin with ATP and purine type lead compounds, molecular dynamics simulations were employed using Gromacs 2021.5^51^. The Amber-ff99SB force field parameters were employed to model the protein, whereas the Generalized Amber Force Field (GAFF) was utilized for the lead compounds, following a similar approach as outlined in previous studies^52,53^. The ‘xleap’ module of AmberTools22 was employed to set up the simulation systems^54^. A cubic periodic box measuring 10 Å on each side was used, and the necessary Sodium (Na+) ions were added to balance the system charge. Next, the ‘ParmEd tool’ was used to convert ‘prmtop’ and ‘inpcrd’ files into Gromacs-compatible ‘top’ and ‘gro’ files, following a methodology outlined in prior studies^55^. The simulation complexes were subjected to energy minimization through both the steepest descent (5000 steps) and conjugate gradient (2000 steps) methods^51^. To reach equilibrium, the simulated systems went through 1 ns of NVT and NPT simulation. Subsequently, production MD simulations lasting 500 ns were conducted for all systems. Long-range electrostatic interactions were calculated using the particle mesh Ewald method, employing a cut-off distance of 1.0 nm, a Fourier spacing of 0.16 nm, and an interpolation order of 4. The H-bond length constraints are applied using the LINCS algorithm. Finally, the MD trajectory data were analyzed using Gromacs-provided ‘gmx’ ﻿﻿tools^51^.

## Principal component analysis (PCA)

To investigate the conformational changes of proteins during dynamics simulations, Principal Component Analysis (PCA) was employed. The GROMACS tools g_covar and g_anaeig were utilized to compute PCA specifically based on the positional data of Cα atoms. Two distinct analyses were conducted: the first involved calculating atomic displacement covariance matrices, eigenvalues, and eigenvectors using g_covar; the second utilized g_anaeig to project molecular dynamics trajectories onto the resulting eigenvector bases. The protein’s motion was determined by projecting onto the first two eigenvectors (ev1 vs. e2), capturing the most significant movements^56^. Ultimately, the findings were visualized via a 2D plot generated using the XMGrace tool available at https://plasma-gate.weizmann.ac.il/Grace/.

## Free Energy Landscape (FEL)

The collective variable free energy landscape (FEL) was constructed by analysing molecular dynamics simulation trajectories, specifically examining the backbone root mean square deviation (RMSD) and the Cα radius of gyration (Rg) through the utilization of the ‘g_sham’ tool in GROMACS^51^. This FEL provides a means to determine the free energy (G) by evaluating the probability distribution of the system’s states. OriginLab software version 2024b^57^ was used to visualize the FEL.

## Hydrogen bond (H-bond) analysis

Hydrogen bond analysis was performed using the GROMACS v2021.5 suite^51^ to quantify the number of hydrogen bonds formed between katanin and lead compounds throughout the molecular dynamics simulation. These hydrogen bonding interactions are essential for molecular recognition and binding affinity^58,59^.

## Binding energy calculations

For evaluating the binding strength between katanin and purine compounds, we performed binding energy calculations utilizing the ‘MMPBSA.py’ script available in the AMBER suite’s gmx_MMPBSA v1.6.3^60^. The final equilibrated 250 ns (250 ns to 500 ns) MD simulation trajectory for each system were used to compute the binding energy. Entropy contributions were excluded in this study due to their computational intensity, following a similar approach used in the previous studies^52,61^. The comprehensive details on the binding energy calculations are mentioned in the earlier studies^52,62^. The per-residue decomposition energy calculations were performed to explore the energy impact of individual residues within 4Å distance of katanin’s active site.

## Results and discussion

### Structure-based virtual screening and hit identification of purine compounds

**Fig. 2:**
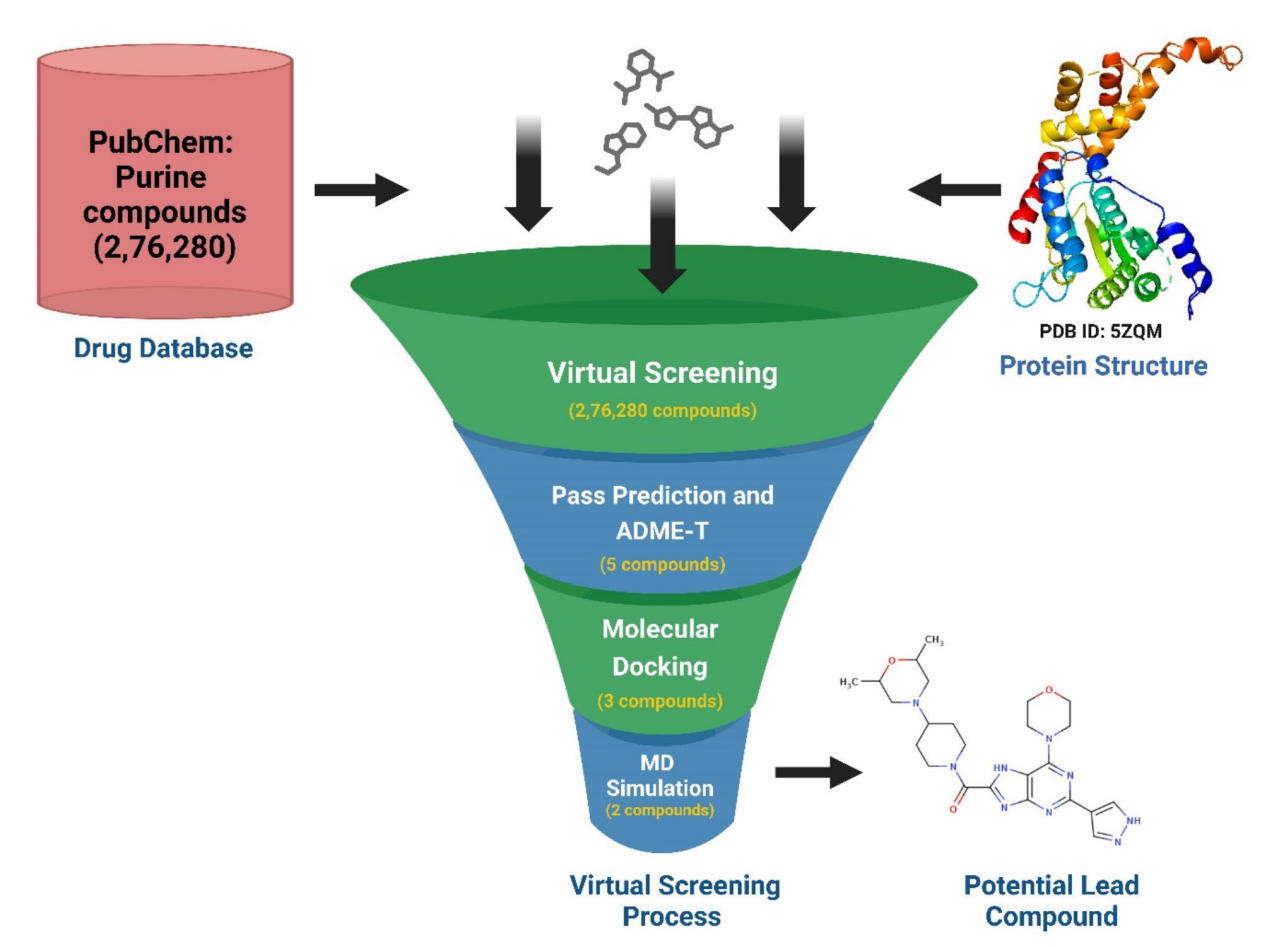
Depicts schematic pipeline for a katanin targeted drug discovery process which begins with a large dataset of 276,280 compounds retrieved from the PubChem database. These compounds were subjected to an initial virtual screening against katanin, a target protein structure (PDB ID: 5ZQM). Subsequent computational steps included pass prediction and ADME-T analysis of top 5 compounds, molecular docking, and molecular dynamics simulations, which progressively filtered the 2 compounds.

**Fig. 3:**
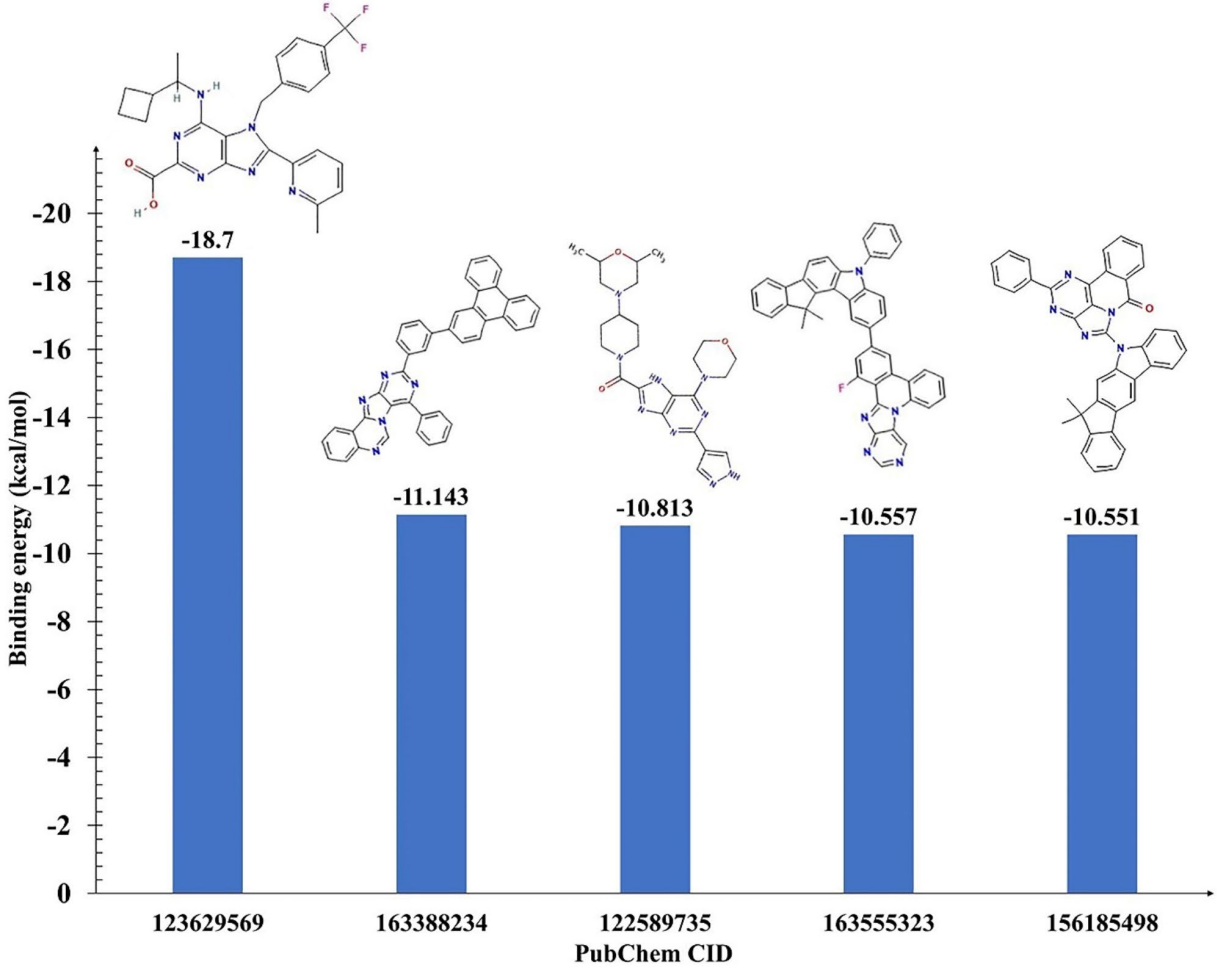
Represents the bar graph of the top five hit compounds based on their binding energies (in kcal/mol) with katanin. The X-axis lists the PubChem CIDs of the compounds, and the Y-axis represents the binding energy. The higher negative value indicates a stronger binding affinity. The corresponding 2D chemical structures of each compound are displayed alongside.

High-throughput virtual screening utilizing molecular docking was conducted on an entire library of purine-type compounds, aiming at the ATP binding pocket of katanin. The objective was to pinpoint purine-types compounds exhibiting a stronger binding affinity, employing AutoDock Vina 1.2.0^42^. Within this screening process, we scrutinized 2,76,280 purine-type compounds, examining their interaction specifically with ATP binding site of katanin, as depicted in Fig. 2. After the initial screening, compounds were sieved based on their binding affinity, culminating in the selection of the top 5 hit compounds exhibiting the lowest binding energy greater than ≤ −10 kcal/mol, suggesting a strong interaction with the ATP binding site of katanin making them promising candidates for further exploration as outlined in Fig. 3. This selection process was executed through InstaDock v1.0^43^. The notable binding affinity exhibited by these top 5 compounds led us to investigate their potential for the development of drugs. To assess the viability of selected compounds, we conducted physicochemical, toxicity, and biological characteristics using ADME-T and pass prediction methodologies.

### PASS prediction and ADME-T properties

The selected top 5 compounds (PubChem CIDs: 123629569, 163388234, 122589735, 163555323, 156185498) underwent pass prediction to check its biological activity prediction by using Way2drug web server^44^. The selected purine-rich compounds from the PubChem database along with their PubChem CID, and 2D structures are mentioned in Fig. 3. Here, all the compounds exhibited Pa values greater than Pi values (Table 1), suggesting their active nature and they may exhibit the biological activity^44,45^. Moreover, all these compounds demonstrated anti-neoplastic and anti-metastatic activity and anti-cancer activity as listed in Table 1. The selected screened compounds showed biological activity as anti-cancer for prostate cancer, non-Hodgkin’s lymphoma, and lymphocytic leukemia as shown in Table 1. These predictions align with the initial goal of identifying katanin inhibitors with potential for cancer treatment. Overall, the PASS predictions offer encouraging preliminary data suggesting the identified purine-type compounds hold promise for katanin inhibition and may have anti-cancer activity.

**Table 1:** Biological activity of the selected hit compounds using the Way2drug webserver.

Sr. No.	PubChem CID	Pa	Pi	Properties
1	123629569	0.213	0.117	Proto-oncogene tyrosine-protein kinase Fgr inhibitor
		0.140	0.102	Liver fibrosis treatment
		0.254	0.092	Cystic fibrosis treatment
2	163388234	0.315	0.038	Prostate cancer treatment
		0.438	0.091	Antineoplastic: sarcoma, lymphoma
3	122589735	0.302	0.222	Antineoplastic (non-Hodgkin’s lymphoma)
		0.185	0.183	Antimetastatic
		0.184	0.084	Prostate cancer treatment
		0.258	0.044	Antineoplastic enhancer
4	163555323	0.552	0.056	Antineoplastic
5	156185498	0.489	0.005	Antineoplastic enhancer
		0.411	0.020	Prostate cancer treatment
		0.263	0.049	Antineoplastic alkaloid
		0.123	0.076	Antineoplastic: lymphocytic leukemia, bladder cancer, glioblastoma multiforme, lymphoma, glioma

The top five compounds also underwent pharmacokinetic as well as toxicology predictions using SWISS-ADME^46^ and pkCSM webserver^47^, respectively. The molecular weight of all the compounds was > 400 kDa indicating stability. Four compounds showed greater lipophilicity indicated by a larger logP value, which implies that the substance has a stronger propensity to diffuse into lipid-rich environments like cell membranes. This can be advantageous for drug delivery and tissue penetration. Water solubility criteria were categorized as insoluble (<−10), poorly soluble (−6), moderately soluble (−4), soluble (−2), and very soluble (0), with Esol indicating poor solubility for PubChem CIDs 123629569 and 156185498 and high solubility for PubChem CID 122589735. This is a factor to be considered for drug formulation and administration, suggesting the advantage of PubChem CID 122589735 compound over others. Lipinski’s rule, specifying < 5 hydrogen bond donors, < 10 hydrogen bond acceptors, and molecular weight < 500 Da, showed only one violation, which is acceptable. Bioavailability was predicted as 0.55, indicating neutrality, suggesting good oral absorption. None of the compounds exhibited PAINS (Pan-Assay Interference Compounds), which is a positive indicator of their drug-like properties. Synthetic availability ranged between 1 (very easy) to 10 (difficult), with all compounds scoring < 5 as shown in Table 2, indicating that these compounds can be synthesized relatively easily. The toxicity profiles varied among the compounds. Some exhibited AMES toxicity and hepatotoxicity, while others did not. Overall, the ADME-T analysis indicated that the identified compounds possess promising drug-like properties. After analysing these top 5 compounds concerning their pass prediction and ADME-T properties, we selected top 3 lead compounds PubChem CIDs 123629569, 163388234, and 122589735 for further molecular docking study.

**Table 2:** ADME-T properties of the selected top five compounds.

ADME Parameters	Properties	PubChem CID 123629569	PubChem CID 163388234	PubChem CID 122589735	PubChem CID 163555323	PubChem CID 156185498
**Physiochemical Properties**	Formula	C26H25F3N6O2	C67H44F3N12O	C24H33N9O3	C44H28FN5	C39H25N5O
	Molecular weight (g/mol)	510.51	1090.14	495.58	645.73	579.65
Absorption	GI Absorption	Low	High	High	Low	Low
	Water Solubility	Poorly soluble	Insoluble	Soluble	Insoluble	Poorly soluble
Distribution	BBB Permeation	No	No	No	No	No
	Lipophilicity (ILogP)	3.02	0	2.79	4.97	4.84
Metabolism	CYP2D6 Substrate/ Inhibitor	Yes	No	Yes	No	No
Excretion	OCT2 Substrate	No	Yes	No	Yes	Yes
Toxicity	AMES toxicity	No	Yes	No	Yes	Yes
	Maximum Tolerance Dose	0.472	0.438	0.644	0.438	0.438
	Hepatotoxicity	Yes	No	Yes	No	No
	Skin Sensitisation	No	No	No	No	No
Drug likeness and medicinal chemistry	Lipinski	1	3	1	2	2
	Bioavailability score	0.56	0.17	0.55	0.17	0.17
	PAINS	0	0	0	0	0
	Synthetic accessibility	4.15	7.39	4.81	4.22	4.09

### Interaction of katanin with purine type compounds using docking

**Fig. 4:**
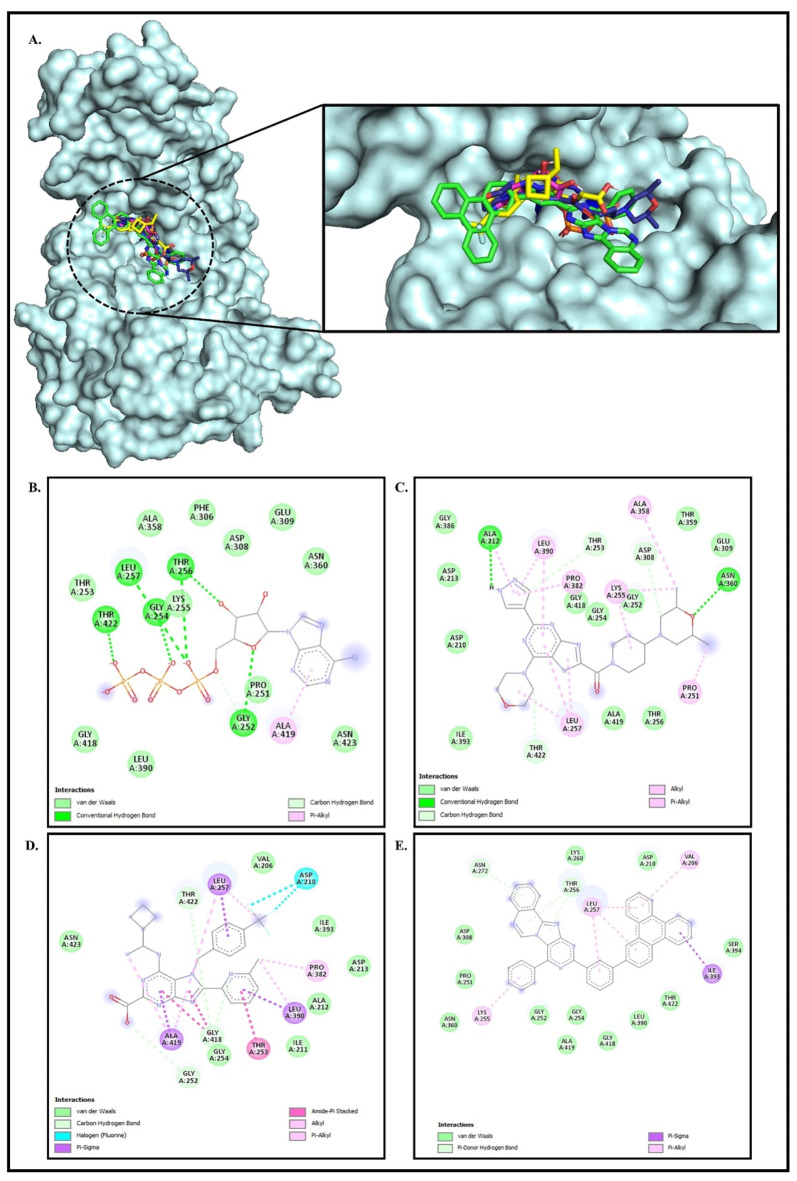
Molecular Docking of Katanin with ATP and Purine-Type Compounds. Panel **(A)** showcases the docked conformation of Katanin (cyan) with ATP (violet) and with lead compounds PubChem CID 122589735 (blue), PubChem CID 123629569 (yellow), and PubChem CID 163388234 (green), located at the ATP binding pocket (residues 141–171) of Katanin along with an enlarged view of binding site. Panel **(B)** illustrates the intricate interaction network of the Katanin-ATP complex. Panel **(C)** highlights 2D interaction network of the Katanin-122589735 complex. Panel **(D)** shows the interaction network with the Katanin-123629569 complex, and Panel **(E)**highlights the 2D interaction network with the Katanin-163388234 complex after docking. The 2D interaction analysis was performed using the Discovery Studio Visualizer^50^.

First, we performed a control docking of katanin with ATP using AutoDock 4.2.7^48^, because it provides valuable insights into the protein’s binding preferences and interactions with its natural substrate. The least energy docked conformation of ATP was found to be −4.86 kcal/mol (Fig. 4B; Table 3). The analysis of Katanin-ATP complex shows that the ATP is stabilized by the bonded and non-bonded type of interactions as shown in Fig. 4; Table 3. ATP forms a conventional hydrogen bonding interaction with residues Gly252 (1.68 and 2.33Å), Thr253 (2.21 Å), Gly254 (2.81 and 2.10 Å), Lys255 (2.52 Å), Thr256 (1.71 and 3.05 Å), Leu257 (2.57 Å), Asp210 (2.41 Å), Thr422 (2.04Å), whereas Leu257 forms a π-sigma, and Leu390 forms π-alkyl type of non-bonded interactions as shown in Fig. 4A and B; Table 3. The docked study showed that the ATP forms stable complex with katanin by forming stronger hydrogen bonding as well as van der Waals, CH and π-alkyl type of interactions as shown in Fig. 4B.

**Table 3:** Analysis of hydrogen bonding interactions of lead compounds with katanin receptor after molecular docking.

PubChem Compound CID	Binding Energy (kcal/mol)	Atoms involved in binding	Bond type	Distance	Angle	Figure
Katanin-ATP	−4.86	GLY252:HN - ATP501:O20 THR253:HN - ATP 501:O1B GLY254:HN - ATP 501:O5’ GLY254:HN - ATP 501:O1B LYS255:HN - ATP 501:O1B THR256:HN - ATP 501:O3A LEU257:HN - ATP 501:O2A ATP 501:H61 - ASP210:O GLY252:CA - ATP 501:O2G THR256:CB - ATP 501:O1A THR422:HG1 - ATP501	H Bond H Bond H Bond H Bond H Bond H Bond H Bond H Bond H Bond H Bond H Bond	1.68 2.21 2.81 2.10 2.52 3.05 2.57 2.41 2.33 1.71 2.04	164.55 119.63 121.04 143.09 133.19 99.24 127.69 143.99 126.59 154.75 107.42	4B
Katanin − 122589735	−8.85	ASN360:ND2 - Drug: O Drug: H - ALA212:O Drug: C - THR422:OG1 Drug: C - ASP308:OD2 Drug: C - THR253:O	H Bond H Bond CH Bond CH Bond CH Bond	3.15 2.50 3.16 3.30 3.38	93.24 - 90.31 125.53 94.84	4 C
Katanin − 123629569	−8.57	GLY252:CA - Drug: O GLY418:CA - Drug: N Drug: C - GLY418:O Drug: C - THR422:OG1	CH Bond CH Bond CH Bond CH Bond	3.66 3.22 3.50 3.19	93.75 105.9 96.9 113.2	4D
^ϯ^Katanin- 163388234	−8.51	-	-	-	-	4E

^ϯ^ There are no conventional and CH-type hydrogen bonding interactions with katanin.

Similarly, molecular docking was performed to explore the binding mode and affinity of selected PubChem CIDs 122589735, 123629569 and 163388234 with katanin using AutoDock 4.2.7^48^. The least binding energy conformation of PubChem CIDs 122589735, 123629569, and 163388234 were found to be −8.85, −8.57, and − 8.51 kcal/mol, respectively as shown in Fig. 4; Table 3. This revealed that these purine type compounds have higher binding affinity with ATP binding pocket of katanin than the ATP molecule (Table 3).

Notably, PubChem CID 122589735 exhibited the lowest binding energy (−8.85) with katanin compared to all other compounds analysed, suggesting a higher binding affinity with the ATP binding site of katanin and may inhibit the enzyme’s activity. Therefore, to delve into the mechanisms of bonded and non-bonded interactions of PubChem CID 122589735 with katanin, we conducted further analyses of docked complexes and have thoroughly discussed our findings. The analysis of the Katanin-122589735 complex (Fig. 4C) shows that stability of the PubChem CID 122589735 compound is attributed to conventional hydrogen bonding interactions with residues Asn360 (3.15 Å), Ala212 (2.50 Å), whereas carbon hydrogen (CH) bonding interactions with residues Thr422 (3.16 Å), Asp308 (3.30 Å), and Thr253 (3.38 Å) as shown in Table 3; Fig. 4C. In addition, PubChem CID 122589735 makes an alkyl type of interaction with Lys255, Leu257 and 390, Ala358, Pro251 and 382, and van der Waals interaction with Asp210, Asp213, Gly252, Gly254, Thr256, Glu309, Thr359, Gly386, Ile393, Gly418 and Ala419 as shown in Table 3; Fig. 4C. The PubChem CID 122589735 achieves stabilization within the ATP-binding pocket of katanin through the formation of two hydrogen bonds and three carbon-hydrogen bond interactions, along with multiple non-bonded interactions with the surrounding residues. These interactions suggest that the compound may potentially interfere with the enzyme’s activity, offering a promising avenue for therapeutic inhibition.

Further analysis of the Katanin-123629569 complex (Fig. 4D) shows that the PubChem CID 123629569 is stabilized due to CH bonding interactions with Gly252 (3.66 Å), Gly418 (3.22 Å and 3.50 Å), and Thr422 (3.19 Å), also the presence of Halogen (fluorine) type interaction with Asp210 (3.42 Å). In addition, PubChem CID 123629569 forms π-Sigma interactions with Leu257, Leu390, and Ala419, Amide-π stacked bonds with Thr253 (Table 3; Fig. 4D). Moreover, Alkyl type of interactions contributed to Pro382, Leu390, Leu257, and Ala419 as shown in Table 3; Fig. 4D. Thus, this compound primarily forms CH bonds and halogen interactions with katanin. In contrast, PubChem CID 123629569 does not form any hydrogen bonding interactions within the ATP-binding pocket of katanin. Consequently, it exhibits a lower binding energy of −8.57 kcal/mol compared to PubChem CID 122589735 (Table 3), indicating relatively weaker binding affinity and potential efficacy as an inhibitor.

Next, the analysis of the katanin-163388234 complex (Fig. 4E) shows that the PubChem CID 163388234 compound forms π-Donor hydrogen bond for Thr256 (3.98 Å and 3.74 Å), and Asn272 (3.69 Å) (Table 3; Fig. 4E), π-Sigma bond with Ile393 (3.82 Å) and π-Alkyl bonds for Leu257 (4.56 Å), Val206 (5.25 Å), Leu257 (5.24 Å and 4.55 Å) as well as Lys255 (5.32 Å) as shown in Table 3; Fig. 4E.

PubChem CID 163388234 lacks both hydrogen bonding and carbon-hydrogen bonding interactions with the ATP-binding pocket of katanin, as it lacks electron bond donor groups such as O, N, S, etc. As a result, it demonstrates a lower binding energy of −8.51 kcal/mol compared to PubChem CID 122589735 and PubChem CID 123629569 (Table 3), suggesting a significantly weaker binding affinity and reduced potential as an effective inhibitor.

The molecular docking results suggest that the PubChem CID 122589735 has the potential to bind to the ATP binding site of katanin as it forms the hydrogen bonding, CH bonding, van der Waals and electrostatic interactions (Fig. 4; Table 3). Hence, to explore the refined binding mode and affinity of katanin with ATP, PubChem CID 122589735, and PubChem CID 123629569, molecular dynamics simulations were employed and discussed in the below section.

### Molecular dynamics (MD) simulations

**Fig. 5:**
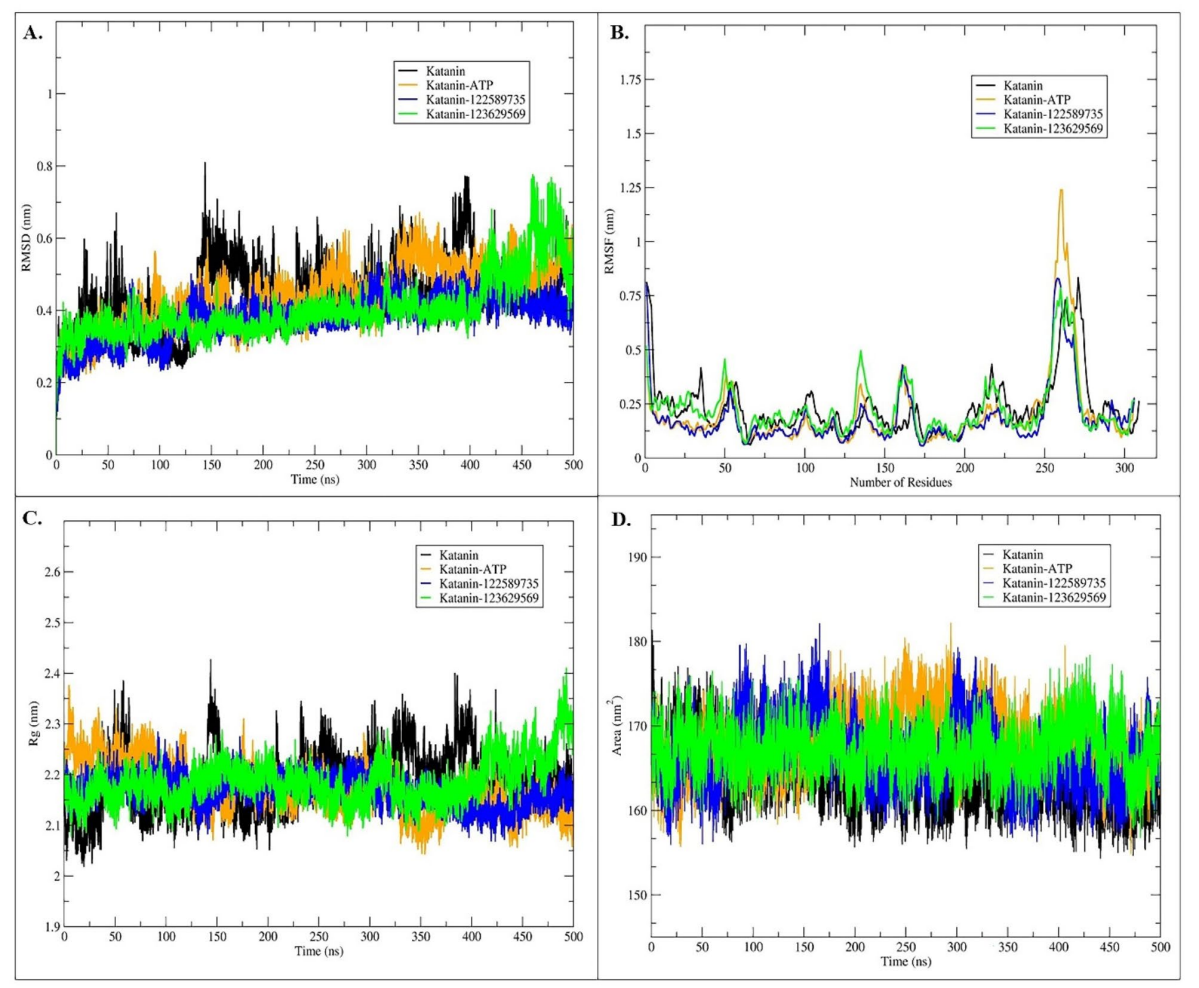
MD simulation analysis of katanin and lead compounds. Katanin is represented in black, the Katanin-ATP complex in orange, whereas the Katanin-122589735 complex and Katanin-123629569 complex are represented in blue and green respectively. Panel **(A)** shows the RMSD plot of C_α_ backbone atoms katanin and Katanin with ATP and lead compounds, **(B)** shows the RMSF plot of C_α_ backbone atoms of katanin, **(C)** represents the Rg, and **(D)** SASA of katanin for 500ns time steps.

MD simulations were performed to investigate the interaction of katanin with ATP, PubChem CID 122589735, and PubChem CID 123629569 using Gromacs 2021.5^51^. The least energy docked conformation of Katanin, Katanin-ATP, Katanin-123629569, and Katanin-122589735 shown in (Fig. 4), were considered as starting conformations for MD simulations. The simulations were performed for 500 ns, to obtain detailed conformational and structural changes in the katanin (Supplementary movies 1–4). The stability of the simulation systems was assessed by plotting the root mean square deviation (RMSD) of the C_α_ backbone atoms of protein (Fig. 5A). RMSD plot revealed that all the simulation systems reached their equilibrium after 300ns, indicating the stability of the katanin-lead compounds complexes (Fig. 5A). Overall, katanin with ATP and drug complexes shows a lower RMSD value compared to katanin in apo form (Fig. 5A), this shows that the ATP and lead compound has a profound effect on the structure and dynamics of katanin (Supplementary movie 1–4). The Katanin-123629569 shows higher fluctuations compared to all other katanin complexes after 300ns (Fig. 5A). To gain more insight into the impact of drug binding and conformational changes in the katanin structure, root mean square fluctuations (RMSF) of C_α_ atoms were performed and shown in Fig. 5B.

It was revealed that katanin bound to ATP and lead compound 122589735 showed lower RMSF (Fig. 5B) compared to the katanin and Katanin-123629569. The ATP binding site residues of katanin (141 to 171) show reduced fluctuations of Katanin-122589735, suggesting a stronger bonded and non-bonded interaction with 122589735 compared to the katanin with ATP and 123629569 (Fig. 5B). Moreover, the Katanin-122589735 complex revealed lower conformational changes in lead compound 122589735 (Supplementary Movie 3) while the PubChem CID 123629569 showed higher conformational fluctuations (Supplementary Movie 4). This might be because PubChem CID 122589735 compound forms two hydrogen bonding interactions with Asn360 and Ala212, along with three CH bonding interactions with Thr422, Asp308, and Thr253 of katanin (Fig. 5B and C; Table 4). In contrast, PubChem CID 123629569 only engages in non-bonded interactions. Altogether, katanin with compound 122589735 shows a profound effect on the structure and dynamics of katanin.

To further check the compactness of the protein, the radius of gyration (Rg) (Fig. 5C) and solvent-accessible surface area (SASA) (Fig. 5D) were calculated. The Rg plot analysis revealed that katanin-ATP and katanin-122589735 complexes exhibit a lower Rg value compared to katanin and katanin-123629569 complexes (Fig. 5C). This suggests that katanin adopts a more compact conformation when bound compound 122589735 (Fig. 5C) and impact the dynamics of katanin. Similarly, the SASA plot complements Rg analysis by focusing on the surface area of the protein accessible to the solvent molecule. The collective SASA values of all the systems ranged between 160 and 180 nm^2^ (Fig. 5D). The findings suggest that the lead compounds, particularly PubChem CID 122589735, can induce significant conformational changes in katanin, potentially affecting its function. These changes might disrupt katanin’s ability to interact with microtubules or other proteins, leading to inhibition of its activity. Additionally, to comprehensively characterize the protein’s conformational changes upon binding to ATP and lead compounds throughout the simulation, we employed a multi-pronged approach which includes principal component analysis (PCA), free energy landscape, hydrogen bonding interaction, and binding energy calculations.

### Analysis of conformational changes using PCA and FEL

PCA was carried out using the gmx_covar and gmx_anaeig modules of Gromacs 2021.5^51^ to understand the essential motions of the katanin, katanin-ATP and katanin-122589735, and katanin-123629569 complexes. The extracted eigenvectors, or principal components (PCs), were analysed to characterize the dominant conformational changes in the katanin. Whereas to explore the energetic landscape underlying these conformational changes, we performed free energy landscape (FEL) analysis. FEL analysis shows the protein conformational space concerning energy and time. The calculated free energy surfaces revealed multiple metastable states corresponding to distinct conformational basins.

**Fig. 6:**
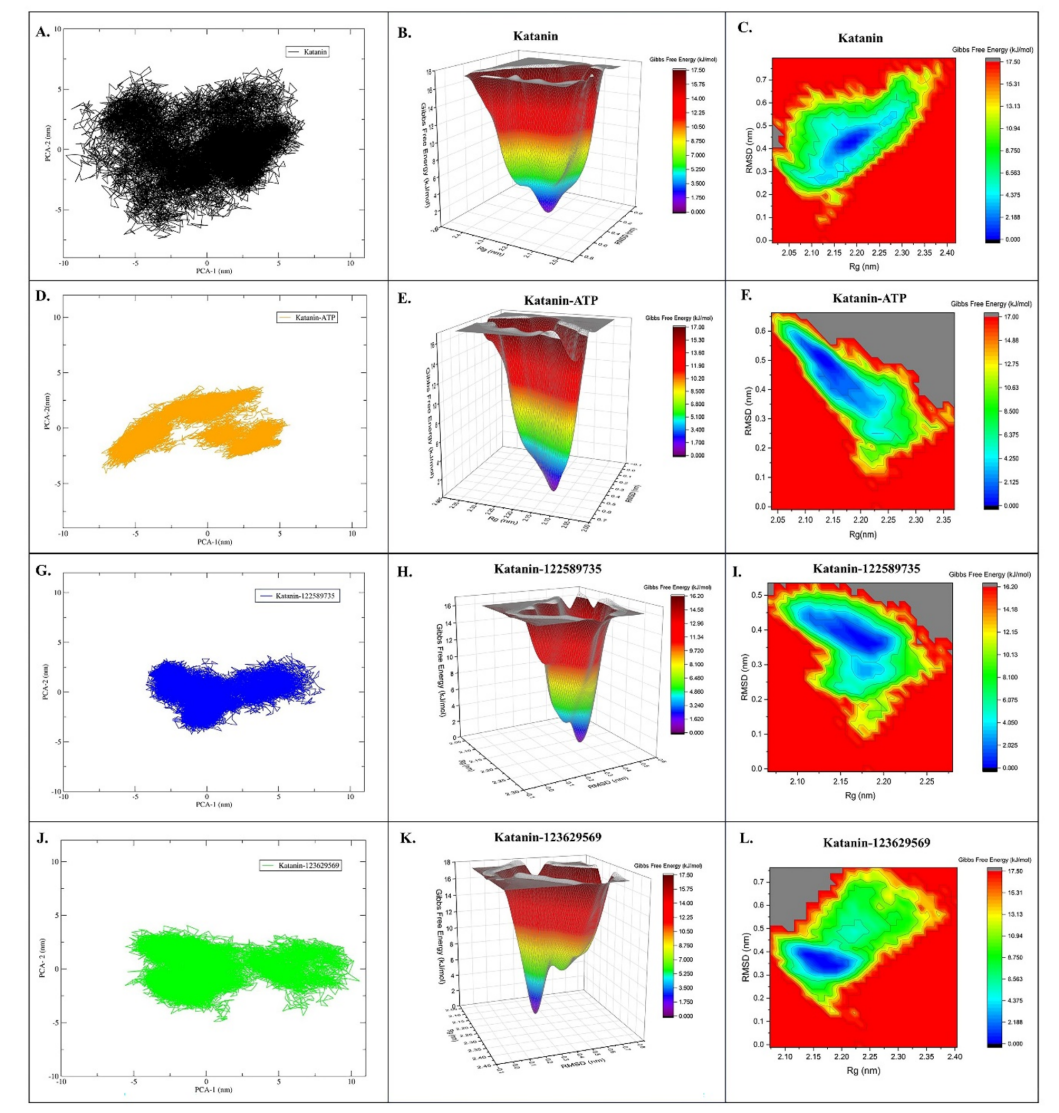
Shows PCA & FEL analysis of katanin and katanin with ATP and lead compounds: Here, panel (**A)** shows the PCA plot and **(B-C)** shows the FEL of katanin, panel (**D)** shows the PCA plot and **(E-F)** shows the FEL of Katanin-ATP complex, panel (**G)** shows the PCA plot and **(H)** and **(I)** shows the FEL of Katanin-122589735 complex, and last panel **(J)** shows the PCA plot and **(K-L)** shows the FEL of Katanin-123629569 complex. The graphs displayed in panels A to L were generated using the built-in *gmx *tool in GROMACS. Panels A, D, G, and J were visualized through XMGrace tool, while panels B, C, E, F, H, I, K, and L were visualized using OriginLab software version 2024b^57^.

The PCA revealed a significant difference in conformational diversity between the unbound katanin and the various complexed states as shown in Fig. 6. The unbound katanin exhibited a higher degree of conformational variability, as evidenced by the broader distribution of PCA scores (PCA2: 7, PCA1: 7) as shown in Fig. 6A. Similarly, in the FEL plot (Fig. 6B), the unbound katanin exhibited a more complex landscape with several interconnected basins, indicating a higher degree of conformational flexibility. Likewise, a greater number of high energy states are observed as seen in the contour map plot of katanin (Fig. 6C).

When katanin was in bound state such as katanin-ATP, katanin-122589735 and katanin-123629569 complexes showed lower conformational diversity with PCA scores (PCA2: 4, PCA1: 5), (PCA2: 4, PCA1: 8) and (PCA2: 4, PCA1: 10), respectively, as shown in Fig. 6D, G and J. This suggests that the binding of ATP or the lead compounds significantly restricts katanin’ s conformational flexibility. To gain further insights into the structural changes associated with these conformational differences, we examined that PC1 and PC2, which together explain a significant portion of the variance of katanin and katanin with lead compounds. These findings suggest that the binding of ATP or the lead compounds induces conformational changes in the katanin, leading to a more stable and restricted state.

The same observation was made for FEL analysis, wherein the bound states displayed a more restricted landscape with a dominant metastable state, suggesting a more stable conformation. Here, the Katanin-122589735 complex had the least energy range between 0 and 1.62 kJ/mol for the deep energy minima (Fig. 6H and I). Whereas the Katanin-123629569 shows single minima but undergoes more variations/transition state in the structural folding as compared to Katanin-122589735 to achieve the least energy state (Fig. 6K and L). These results infer that the Katanin-122589735 complex achieves the global state conformation sooner than the Katanin-123629569 complex.

These observed conformational changes and energetic landscape features have important implications for katanin’ s biological function. The binding of ATP or the lead compounds appears to induce a more stable conformation, potentially affecting katanin’s catalytic activity. Thus, our PCA and FEL analyses together provide a comprehensive picture into the conformational dynamics of katanin and its complexes as shown in Fig. 6. The results suggest that the binding of ATP or lead compounds significantly restricts katanin’s conformational flexibility, potentially influencing its biological function. To gain a deeper understanding of the interactions between katanin and lead compounds, we specifically investigated hydrogen bonding through a dedicated analysis.

### Hydrogen bond interaction analysis

The hydrogen bond analysis provides insights into the stability and dynamics of the protein-ligand interactions during the MD simulations. This analysis quantified and characterized the hydrogen bond formation between katanin and ATP, lead compounds 122589735, and 123629569 during the 500 ns time steps (Supplementary Fig. 2). Katanin-ATP complex shows more stable and frequent hydrogen bond formation, suggesting a strong and specific interaction (Supplementary Fig. 2A). Next, hydrogen bond analysis with the lead compounds shows that both, Katanin-122589735 and Katanin-123629569 form hydrogen bonds, but the stability and frequency differ. The Katanin-122589735 complex (Supplementary Fig. 2B) forms stable hydrogen bonds as compared to the Katanin-123629569 complex (Supplementary Fig. 2C). Whereas the number of hydrogen bonds increases after 300ns for the Katanin-122589735 complex indicating stronger interaction between PubChem CID 122589735 and katanin (Supplementary Fig. 2B). The hydrogen bond analysis shows that compound 122589735 is stable at the ATP binding pocket of katanin as evidenced by the consistent number of hydrogen bonds over time. (Supplementary Fig. 2B). To obtain clear perspective regarding the stronger interactions between katanin and lead compounds, the binding affinity of lead compounds with katanin was investigated. For this we employed the gmx_MMPBSA tool v.1.6.3^60^.

### Binding energy calculations

The binding energy calculations using MM-GBSA provided quantitative insights into the strength of the interactions between katanin and the lead compounds. We employed binding energy calculations using the MM-GBSA method through gmx_MMPBSA v1.6.3 tool^60^. The MD simulated equilibrated trajectory from 250 ns (a total of 2501 frames) were considered for the binding energy calculations.

**Table 4:** Binding energy calculation of katanin with ATP and lead compounds. All energies are in kcal/mol.

Katanin-drug complex	ΔE_vdw_	ΔE_ele_	ΔE_gas_	ΔE_sol_	ΔE_bind_
**Katanin-ATP**	−40.91	151.60	110.69	−138.18	**−27.49**
**Katanin-122589735**	−45.21	−19.43	−64.64	31.88	**−32.76**
**Katanin-123629569**	−39.12	−20.75	−59.87	32.12	**−27.75**

The binding free energy data analysis revealed that katanin had a higher binding affinity with PubChem CID 122589735 compound compared to PubChem CID 123629569 and ATP and as shown in Table 4. The order of binding affinity decreases in the order of PubChem CID 122589735 (−32.76 kcal/mol) > PubChem CID 123629569 (−27.75 kcal/mol) > ATP (−27.49 kcal/mol). This suggests that PubChem CID 122589735 compound forms more higher binding affinity with katanin compared to PubChem CID 123629569 and ATP. Furthermore, the analysis revealed that the van der Waals and electrostatic interactions play a significant role in the binding of the 122589735 compound with katanin whereas, the loss of these interactions reduces the affinity of PubChem CID 123629569 compound for katanin. This suggests that these interactions are essential for the stability and specificity of the katanin-lead compound complexes. Additionally, utilizing decomposition analysis with the gmx_MMPBSA v1.6.3 tool to identify the actively interacting residues at the binding site interface, the per-residue contributions of katanin interacting with the lead compounds and altering binding affinity were ascertained (Supplementary Fig. 3).

The per-residue decomposition analysis of katanin-ATP complex shows 15 highly interacting residues of katanin that are involved in the binding with ATP (Supplementary Fig. 3A). Whereas the Katanin-122589735 complex had 12 active residues contributing to energy and notably, Leu74, Ala236, and Thr239 exhibited the highest energy contributions (Supplementary Fig. 3B). In the Katanin-123629569 complex, Leu74, Leu207 and Ala236 were found to have significant roles in drug binding (Supplementary Fig. 3C). Overall, residue decomposition analysis suggests that residues in the ATP binding site, such as Thr70, Gly71, Leu74, Gly235, and Ala236 are common active site residues of katanin that interact with both ATP and lead compounds (Supplementary Fig. 3A-3C). This suggests that these residues are critical for the interaction with lead compounds at the ATP binding site. While some residues are common to both ATP and the lead compounds, other residues exhibit unique contributions to the binding of each lead compound. For example, Ile28, Lys72, and Thr73 contributes highly to the ATP binding, while Leu207 and Ser211 contribute significantly to the binding of PubChem CID 122589735. Given that both docking and per-residue analysis focused on the ATP binding site, residues within this region such as Thr70, Gly71, Leu74, Gly235, Ala236 are likely to be consistently involved in interactions with both ATP and the lead compounds as shown in Fig. 4 and Supplementary Fig. 3. Overall, the per-residue decomposition analysis provided valuable insights into the structural determinants of lead compounds binding to katanin. This information can serve as a valuable resource for guiding the development and refinement of innovative katanin inhibitors, offering a promising outlook for future advancements.

## Conclusion

This study identified potential purine-type inhibitors targeting the ATP binding pocket of microtubule-severing enzyme, katanin which plays essential role in various carcinoma. Screening the PubChem database revealed promising lead candidates, including PubChem CIDs 122589735 and 123629569, which demonstrated significant binding affinities with katanin. Among these, PubChem CID 122589735 emerged as the most promising candidate, exhibiting a superior binding energy of −32.76 kcal/mol compared to ATP (−27.49 kcal/mol) and PubChem CID 123629569 (−27.75 kcal/mol) (Table 4). This lead compound holds potential for drug repurposing and katanin inhibition, highlighting its therapeutic promise. To validate these computational predictions and advance their clinical applicability, further experimental investigations are essential. Overall, this computational modelling study not only identifies a potent katanin inhibitor but also establishes a foundation for developing innovative therapeutic strategies against various carcinomas.

## Electronic supplementary material

Below is the link to the electronic supplementary material.

Supplementary Material 1

Supplementary Material 2

Supplementary Material 3

Supplementary Material 4

Supplementary Material 5

## Data Availability

Data is provided within the manuscript or supplementary information files.
