# Supplementary material for: Exploring purine analogues as inhibitors against Katanin, a microtubule severing enzyme using molecular modeling approach
Source: Sci Rep. 2024 Dec 30;14:32095. doi: 10.1038/s41598-024-83723-7 (PMC11686324; doi:10.1038/s41598-024-83723-7)
Supplement: Supplementary file 5 — Supplementary Material 5 [file 41598_2024_83723_MOESM5_ESM.docx]

**Supplementary Information**

**Exploring Purine Analogues as Inhibitors against Katanin, a Microtubule Severing Enzyme using Molecular Modeling Approach**

Vibhuti Saxena, Pruthanka Patil, Purva Khodke, and **Bajarang Vasant Kumbhar***

Department of Biological Sciences, Sunandan Divatia School of Science, NMIMS (Deemed to be) University, Vile Parle (West), Mumbai- 400056, India

**Equal Authors: Vibhuti Saxena and Pruthanka Patil**

***Corresponding Author**

**Dr. Bajarang Vasant Kumbhar**

Assistant Professor

Sunandan Divatia School of Science

Department of Biological Science

NMIMS (Deemed to be) University, Mumbai

Maharashtra-400056, India

Contact: (91-22) 4235 5963

Email: [bajarang.kumbhar@nmims.edu](mailto:bajarang.kumbhar@nmims.edu), [kumbharbajarang@gmail.com](mailto:kumbharbajarang@gmail.com)

**Supplementary Figures**


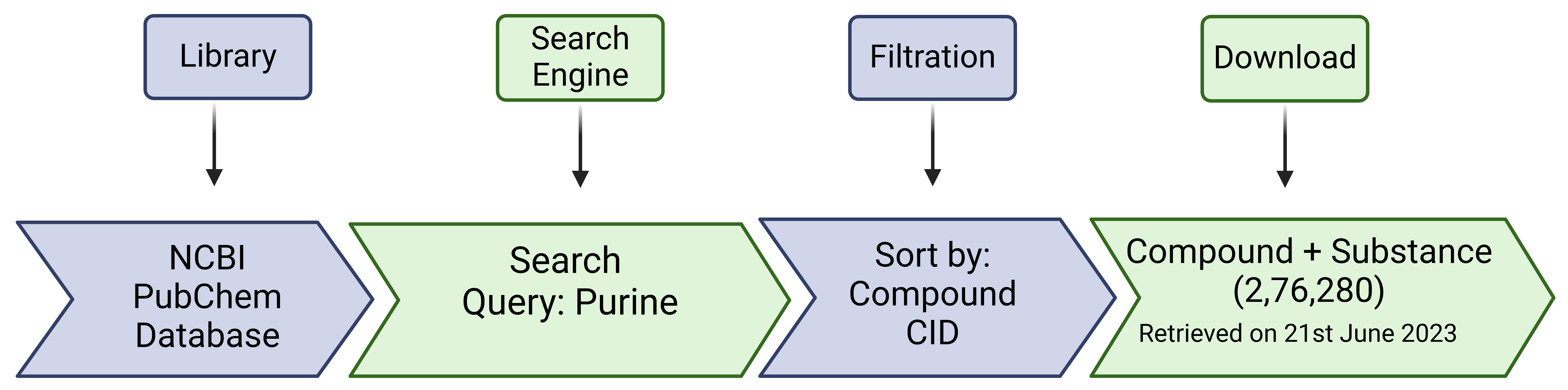
**Supplementary Figure 1: Schematic representation of the data retrieval from the NCBI PubChem database.** Firstly, by utilizing the search query as Purine which triggers a search within the database for compounds and substances related to it is done. The results are then refined through a Filtration process, sorting them by compound and compound identifier (CID) which was further downloaded, indicating the successful retrieval and storage of the relevant information as our own purine inhibitor library.



**Supplementary Figure 2: Hydrogen bond analysis of Katanin with ATP and drug complexes.** Plot **(A)** illustrates Katanin-ATP complex (orange), Plot **(B)** shows Katanin-122589735 complex (blue) and Plot **(C)** shows Katanin-123629569 complex (green).


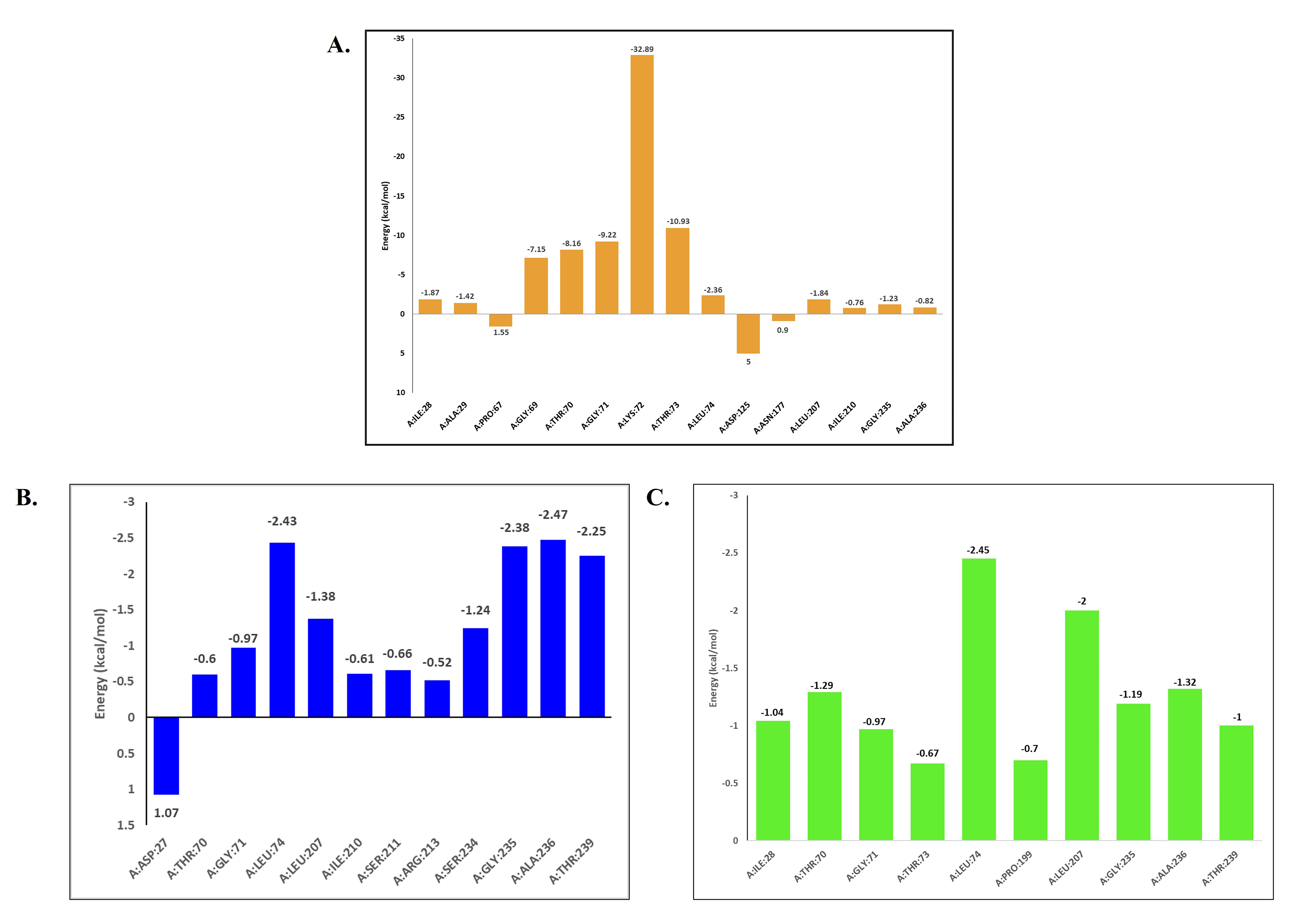
**Supplementary Figure 3: Per-residue energy decomposition analysis.** Plot **(A)** shows the energy contribution between Katanin and ATP which shows 15 highly interacting residues such as Lys72, Thr23 and Gly71, etc. Plot **(B)** shows katanin-122589735 complex which has 12 active residues contributing to energy which includes Leu74, Ala236, and Thr239 residues. Plot **(C)** shows katanin-123629569 complex where residues like Leu74, Leu207 and Ala236 are actively involved in energy contribution.

**Supplementary Movie 1.** MD simulation of katanin in apo form.

**Supplementary Movie 2.** MD simulation of katanin with ATP.

**Supplementary Movie 3.** MD simulation of katanin with 122589735 lead compound

**Supplementary Movie 4.** MD simulation of katanin with 123629569 lead compound
